# Development of 6′-*N*-Acylated Isepamicin Analogs with Improved Antibacterial Activity against Isepamicin-Resistant Pathogens

**DOI:** 10.3390/biom10060893

**Published:** 2020-06-11

**Authors:** Yeon Hee Ban, Myoung Chong Song, Hee Jin Kim, Heejeong Lee, Jae Bok Wi, Je Won Park, Dong Gun Lee, Yeo Joon Yoon

**Affiliations:** 1 Natural Products Research Institute, College of Pharmacy, Seoul National University, Gwanak-gu, Seoul 08826, Korea; yhban@snu.ac.kr (Y.H.B.); smch517@snu.ac.kr (M.C.S.); 2 Department of Chemistry and Nanoscience, Ewha Womans University, Seoul 03760, Korea; kimijini93@gmail.com; 3 School of Life Sciences, BK21 Plus KNU Creative BioResearch Group, College of Natural Sciences, Kyungpook National University, Daehakro 80, Bukgu, Daegu 41566, Korea; gml09wjd@naver.com (H.L.); dglee222@knu.ac.kr (D.G.L.); 4 Department of Integrated Biomedical and Life Sciences, Korea University, Seoul 02841, Korea; slwlwhs@korea.ac.kr (J.B.W.); jewonpark@korea.ac.kr (J.W.P.)

**Keywords:** isepamicin analogs, 6′-*N*-acylation, enzymatic synthesis, antibacterial activity, cytotoxicity

## Abstract

The development of new aminoglycoside (AG) antibiotics has been required to overcome the resistance mechanism of AG-modifying enzymes (AMEs) of AG-resistant pathogens. The AG acetyltransferase, AAC(6′)-APH(2″), one of the most typical AMEs, exhibiting substrate promiscuity towards a variety of AGs and acyl-CoAs, was employed to enzymatically synthesize new 6′-*N*-acylated isepamicin (ISP) analogs, 6′-*N*-acetyl/-propionyl/-malonyl ISPs. They were all active against the ISP-resistant Gram-negative bacteria tested, and the 6′-*N*-acetyl ISP displayed reduced toxicity compared to ISP in vitro. This study demonstrated the importance of the modification of the 6′-amino group in circumventing AG-resistance and the potential of regioselective enzymatic modification of AG scaffolds for the development of more robust AG antibiotics.

## 1. Introduction

**Figure 1:**
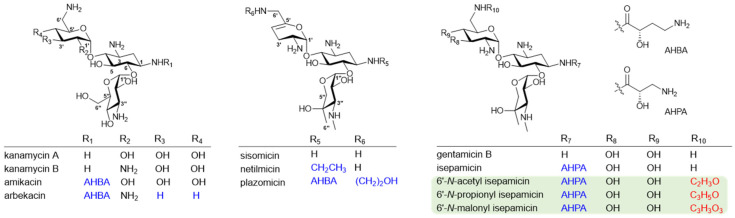
Chemical structures of aminoglycoside (AG) antibiotics currently used in the clinic and 6′-*N*-acylated isepamicin (ISP) analogs developed in this study. Kanamycin A, kanamycin B, sisomicin, and gentamicin B are natural products, whereas amikacin, arbekacin, netilmicin, plazomicin, and ISP are semi-synthetic AGs, in which the chemically modified features are indicated by blue colors. The greenish inset depicts newly synthesized 6′-*N*-acylated ISP analogs and red colors represent the functional group formed by the AAC(6′)-APH(2″)-catalyzed enzymatic synthesis in this study.

Aminoglycosides (AGs), one of the oldest classes of antibiotic agents, have a strong antibacterial activity against Gram-positive and Gram-negative bacterial pathogens because they interfere with the protein biosynthesis by acting on the bacterial ribosome [1,2]. However, a serious antimicrobial resistance has emerged as a result of the long-term and wide clinical use of AGs [2,3]. The primary resistance mechanism is the chemical modification of AG structures by AG-modifying enzymes (AMEs) [2,4,5]. This led to substantial efforts to develop semi-synthetic AGs that can circumvent relevant AMEs and improve the pharmacological profile. For example, amikacin, arbekacin, isepamicin (ISP), netilmicin, and plazomicin were specifically engineered to be less susceptible to the action of clinically important AMEs (Figure 1) [4,5]. The semi-synthetic modification of AGs could still be an efficient approach for the development of more robust new antibacterial drugs against AG-resistant pathogens.

The diverse structural motifs of AG scaffolds can be modified for the development of new antibiotics with improved biological activities and reduced toxicity [6,7]. *N*-acyl moieties at C1 or C6′ positions are the common structures of the semi-synthetic antibiotics currently employed clinically, including the most recently approved antibiotic, plazomicin [8]. AG analogs with a *N*-acyl moiety possess significant pharmacological advantages, but the desired chemical modification at the specific amine position is often impractical due to their structural complexity [6].

The enzymatic synthesis could be an alternative approach for the development of next-generation antibiotics. Butirosin has an (*S*)-4-amino-2-hydroxybutyric acid (AHBA) substituent at the C1-amine, which is introduced by the acyltransferase BtrH and its partner BtrG. The unique AHBA moiety improves antibacterial properties and protects the AG from several common resistance mechanisms [4,5]. Hence, the substrate tolerance of BtrH and BtrG was exploited to successfully introduce an AHBA side chain regiospecifically onto the non-native AGs, suggesting the possibility of the enzymatic synthesis of novel AGs attached with the AHBA moiety [9,10]. Other enzymatic approaches to generate *N*-acylated AGs using AMEs have been reported. Two AG acetyltransferases (AACs), including 3-*N*-AG acetyltransferase AAC(3) and AAC(6′)-APH(2″) with 6′-*N*-acetyltransferase and 2″-*O*-phosphotransferase activities, exhibited promiscuity towards a number of AGs, as well as diverse acyl-CoAs, resulting in the generation of novel mono- and hetero-di-*N*-acylated AGs [11,12,13]. However, the biological activities of most resulting analogs against resistant bacterial pathogens have not been reported. Therefore, the preparation and biological evaluation of more diverse analogs of clinically important AGs are required to prove the potential of using AMEs for the development of therapeutically improved AG agents.

This work reports the new analogs of ISP, a semi-synthetic gentamicin B with a 1-*N*-(*S*)-4-amino-2-hydroxypropionic acid (AHPA) side chain, appending a variety of acyl chains at the C6′-amine position by the enzymatic reaction of AAC(6′)-APH(2″). The antibacterial activity and cytotoxicity of newly synthesized ISP analogs were evaluated. This study demonstrates that the AME-mediated regiospecific modification provides an efficient access to unnaturally acylated AGs with an improved pharmacological potential.

## 2. Materials and Methods

### 2.1. Materials

The ISP was obtained from Toku-E (Bellingham, WA, USA). Acyl-CoAs (acetyl-CoA, propionyl-CoA, malonyl-CoA, butyryl-CoA, crotonyl-CoA, methylmalonyl-CoA, and isovaleryl-CoA), ampicillin, isopropyl *β*-d-1-thiogalactopyranoside (IPTG), hydrochloric acid (HCl), trifluoroacetic acid (TFA), deuterium oxide (D_2_O), and 5-mm Shigemi advanced NMR microtubes were purchased from Sigma-Aldrich (St Louis, MO, USA). The heptafluorobutyric acid (HFBA) and formic acid were obtained from Fluka (St Louis, MO, USA). The HPLC-grade acetonitrile (MeCN), methanol (MeOH), and water were acquired from JT Baker (Philipsburg, NJ, USA). The ODS SPE cartridge (Bond Elut-C18, 3 mL/200 mg) was purchased from Agilent Technologies (Santa Clara, CA, USA). A sample preparation of the SPE cartridge (Sep-Pak^®^ C18 3 cc Vac cartridge, 200 mg Sorbent), Xselect^®^ CSH column XP (2.1 × 100 mm, 2.5 μm), leucine enkephalin, and vacuum manifold were products of Waters Inc. (Milford, MA, USA). *Escherichia coli* BL21 (DE3) and plasmid pET-21c(+) (Novagen, Madison, WI, USA) were used for expression of the recombinant protein. The Ni-nitrilotriacetic acid (NTA) agarose (Qiagen, Valencia, CA, USA), PD10 column (GE Healthcare, Piscataway, NJ, USA), and an Amicon Ultracel 10 K molecular weight cut-off spin filter (Millipore, Bedford, MA, USA) were used to prepare the histidine-tagged recombinant protein.

### 2.2. Overexpression and Purification of AAC(6′)-APH(2″)

The *aac(6′)-aph(2″)* gene derived from *Staphylococcus aureus* (GenBank accession no. NC_002774) was codon-optimized for the expression in *E. coli* and the DNA fragment containing the restriction enzyme sequences (*Nde*I and *Xho*I) was synthesized by DNA 2.0 (Menlo Park, CA, USA). The synthesized DNA fragment was cloned into pET-21c(+) to encode a histidine-tagged construct. For the expression and purification of AAC(6′)-APH(2″), the expression plasmid was introduced into *E. coli* BL21(DE3). The recombinant strain was grown in a LB medium with 50 µg/mL of ampicillin. Each liter of culture was inoculated with 10 mL of an overnight starter culture. The culture was grown at 37 °C to an optical density (OD_600_) of 0.6, then overexpression was induced by 0.5 mM of IPTG for another 3 h.

Cells were harvested by centrifugation (2 min at 20,000 rpm), re-suspended in a lysis buffer (300 mM NaCl, 10 mM imidazole, 50 mM sodium phosphate, pH 8.0), and then lysed by sonication for 5 min using a 2 s on/2 s off cycle. The lysate was clarified by centrifugation (30 min at 20,000 rpm), and the clarified cell lysate was passed through a column of NTA Agarose. After washing the column with a washing buffer (300 mM NaCl, 40 mM imidazole, 50 mM sodium phosphate, pH 8.0), the histidine-tagged protein was eluted with an elution buffer (300 mM NaCl, 500 mM imidazole, 50 mM sodium phosphate, pH 8.0). Imidazole was removed from the purified recombinant protein solution using a PD10 column with a Tris buffer (20 mM Tris-HCl, 250 mM NaCl, 10% glycerol, pH 7.9). The purified AAC(6′)-APH(2″) was concentrated using an Amicon Ultracel 10 K molecular weight cut-off spin filter, and then stored at −80 °C. The sodium dodecyl sulfate-polyacrylamide gel electrophoresis (SDS-PAGE) analysis was employed to ascertain the purity of the protein. The protein concentration was determined by the Bradford protein assay using the bovine serum albumin as a standard. Typically, approximately 10 mg of purified AAC(6′)-APH(2″) was obtained from 1 L of culture (Figure S1).

### 2.3. Chemoenzymatic Synthesis of 6′-N-Acylated ISP Analogs

6′-*N*-acylation by AAC(6′)-APH(2″) was performed in 50 mM of a MOPS buffer (pH 6.5) containing 100 µM ISP, 20 µM acyl-CoA (acetyl-CoA, propionyl-CoA, malonyl-CoA, butyryl-CoA, crotonyl-CoA, methylmalonyl-CoA, or isovaleryl-CoA), and 5 µM AAC(6′)-APH(2″) at 37 °C for 12 h. For the production of new ISP analogs by a large scale enzyme reaction, purified AAC(6′)-APH(2″) (500 µM) was incubated with 20 mM ISP and 2 mM acyl-CoA under the same conditions described above. The reaction was quenched with 0.7% of HFBA and centrifuged at 13,000 rpm for 10 min. The resulting supernatant containing the target product was extracted using an ODS SPE cartridge. The ODS cartridge was conditioned with 0.7% of an HFBA solution (elution with the following as: MeOH 3 mL → 50% *aq.* MeOH (0.7% HFBA) 3 mL → 0.7% HFBA 3 mL), and then the supernatant containing 0.7% of HFBA was loaded and eluted with the following conditions: 3 mL of 5% *aq.* MeOH (0.7% HFBA), 3 mL of 10% *aq.* MeOH (0.7% HFBA), and 3 mL of MeOH. MeOH eluents were dried in vacuo and dissolved in 200 μL of distilled water, and then subjected to the UPLC-qTOF-HR-MS analysis. A further reaction was performed until the substrate was converted completely. 

### 2.4. UPLC-qTOF-HR-MS Analysis and Structural Identification of New ISP Analogs

Each new ISP analog was identified with a Waters XEVO^®^ G2-S qTOF mass spectrometer coupled with a Waters Acquity UPLC^®^ system equipped with a Xselect^®^ CSH column XP consisting of an Acquity I-Class system. A gradient elution using solvent A (50 mM ammonium-TFA, pH 2.0) and solvent B (50% *aq.* MeCN with 50 mM ammonium-TFA, pH 2.0) as the mobile phase at a flow rate of 0.2 mL/min at 40 °C was applied. The MS system was operated in ESI with a positive ionization mode. The typical operating parameters were as follows: Analyzer, resolution mode; capillary voltage (volt.), 3.0 kV; sampling cone volt., 30 V; source temperature (temp.), 120 °C, source offset temp., 80 °C; desolvation temp., 300 °C; cone gas flow, 10 L/h; desolvation gas flow, 600 L/h; helium collision gas. The analyzer was operated with an extended dynamic range at 60,000 resolution (FWHM at *m/z* 556) with an acquisition time of 0.1 s. Leucine enkephalin (400 pg/μL, 50% *aq.* MeCN with 0.1% formic acid) as a lockspray was infused at a rate of 5 μL/min for mass correction. Mass spectra were acquired with a scan range of 50–800 amu with a scan time of 0.1 s. For the MS/MS analysis, helium collision gas was introduced in accordance with the manufacturer’s recommendations. The MassLynx V4.1 software (Waters) was used for instrument control, acquisition, and sample analysis (Waters Co., Milford, CT, USA).

The converted ISP samples were applied to a Dowex^®^ MAC-3 cation exchange column that was conditioned previously with 100 mL of 0.2 N NaOH and 800 mL of neutralized water. Isolation of converted ISP analogs using the cation exchange resin was performed by elution with 0.1–0.12 N of a NaOH buffer. Then, each eluent of converted ISP was subjected to a Sep-Pak^®^ C18 Vac cartridge and eluted with the HFBA solution.

NMR spectra of new ISP analogs were obtained using a Varian INOVA 500 spectrometer (Palo Alto, CA, USA) operating at 500 MHz for ^1^H and 125 MHz for ^13^C nuclei and a Jeol JNM 500 ECZR spectrometer (Tokyo, Japan) operating at 500 MHz for ^1^H and 125 MHz for ^13^C nuclei. Samples for the NMR analysis were prepared by dissolving each compound in 250 μL of D_2_O and placing the solutions in 5 mm of Shigemi advanced NMR microtubes matched to the solvent. All NMR data processing was done using the Mnova software (Mestrelab Research S.L., Santiago de Compostela, Spain).

### 2.5. Antibacterial Susceptibility Test

*E. coli* (ATCC 25922) and *Pseudomonas aeruginosa* (ATCC 27853) were obtained from the American Type Culture Collection (ATCC; Manassas, VA, USA). Antibiotic-resistant *E. coli* strains for this study were provided by the National Biobank of the Kyungpook National University Hospital (KNUH; Daegu, Korea), a member of the KoreaBiobank Network-KNUH. They were obtained (with informed consent) under the institutional review board (IRB)-approved protocols. Five clinical multi-drug resistant *P. aeruginosa* (MDRPA) isolates were obtained from patients at the KNUH [14]. The isolates were identified using the API20NE kit (BioMerieux, Marcy-l’Etoile, Lyon, France) [15].

Bacteria were cultured in the LB medium and the cell suspensions were adjusted to obtain standardized populations by measuring the turbidity with a spectrophotometer (DU530; Beckman, Fullerton, CA, USA). The bacterial strains at an exponential phase (1 × 10^6^ cells/mL) were inoculated and 100 μL of the LB medium were dispensed per well into 96-well microtiter plates. Susceptibility tests were performed by a two-fold standard broth-microdilution of the test compounds, following the Clinical and Laboratory Standards Institute (CLSI) guideline. After 18 h of incubation at 37 °C, the minimal concentration required to prevent the growth of 90% of a given test organism was defined as the minimum inhibitory concentration (MIC_90_). The growth was assayed with a microtiter ELISA Reader (BioTek ELx800; BioTek Instruments Inc., Winooski, VT, USA) by monitoring the optical density at 600 nm [16]. The breakpoint for susceptibility to ISP was defined by an MIC less than or equal to 8 μg/mL [17].

### 2.6. In Vitro Cytotoxicity Assay against Mammalian Renal Cell Lines

To examine the in vitro cytotoxicity of three ISP analogs, a series of MTT-based cell toxicity assays were carried out using three kidney-derived mammalian cell lines from ATCC, i.e., HEK-293 (human), LLC-PK1 (pig), and A-498 (human) as previously described [18]. The concentration of half-maximal lethal dose for cells (LC_50_) was estimated by fitting concentration response curves to the data obtained from at least two independent experiments, using the SigmaPlot software package 10.0.1 (Systat Software Inc., San Jose, CA, USA). Data were expressed as means (*n* = 3) ± standard deviations and tested for significance using the paired or unpaired two-tailed *t*-test with analysis of variance as appropriate. Results with *p* < 0.05 were considered significant.

## 3. Results and Discussion

### 3.1. 6′-N-Acylation of ISP by the AAC(6′)-APH(2″) Enzyme

**Figure 2:**
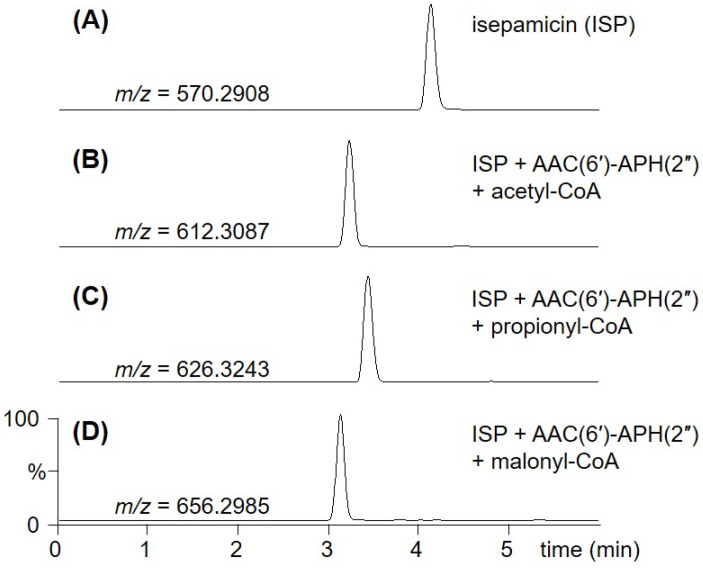
UPLC-qTOF-HR-MS analysis of the ISP and its analogs produced by the enzymatic synthesis using AAC(6′)-APH(2″). (**A**) UPLC-qTOF-HR-MS chromatogram of ISP (selected *m/z* 570.2908). (**B**) UPLC-qTOF-HR-MS chromatogram of A-ISP (selected *m/z* 612.3087) produced by AAC(6′)-APH(2″) enzyme reaction supplemented with acetyl-CoA. (**C**) UPLC-qTOF-HR-MS chromatogram of P-ISP (selected *m/z* 626.3243) produced by AAC(6′)-APH(2″) enzyme reaction supplemented with propionyl-CoA. (**D**) UPLC-qTOF-HR-MS chromatogram of M-ISP (selected *m/z* 656.2985) produced by AAC(6′)-APH(2″) enzyme reaction supplemented with malonyl-CoA.

It is known that the substrate promiscuity of AAC(6′)-APH(2″) allows a diverse modification of the specific amine positions on several AG scaffolds, including the AHBA-attached amikacin and arbekacin [11,13]. However, its activity toward ISP, which has an AHPA side chain at the C1-amine instead of AHBA and pentose sugar as a third ring instead of hexose, has not been tested. Therefore, we first opted to examine the specificity of AAC(6′)-APH(2″) towards ISP and a range of acyl-CoAs. The 58-kDa AAC(6′)-APH(2″) derived from *S. aureus* was expressed in *E. coli* and purified (Figure S1). After the C-terminal hexahistidine tagged AAC(6’)-APH(2”) was incubated overnight with ISP in the presence of a variety of acyl-CoAs, the formation of *N*-acylated ISP was monitored using UPLC-qTOF-HR-MS. No derivative of ISP could be identified when butyryl-CoA, crotonyl-CoA, methylmalonyl-CoA, and isovaleryl-CoA were used as acyl donors. However, incubation with acetyl-CoA, propionyl-CoA, and malonyl-CoA as cosubstrates of AAC(6′)-APH(2″) led to the formation of new UPLC peaks corresponding to the 6′-*N*-acylated products, 6′-*N*-acetyl ISP (A-ISP), 6′-*N*-propionyl ISP (P-ISP), and 6′-*N*-malonyl ISP (M-ISP), at conversion yields of approximately 100%, 100%, and 25%, respectively (Figure 1 and Figure 2). It was reported that the same enzyme could also act on amikacin to produce the 6′-*N*-acety/-propionyl/-malonyl amikacin by 6′-*N*-acylation [11]. Interestingly, the AAC(6′)-APH(2″) enzyme exhibited nearly equal catalytic efficiencies for ISP and amikacin when the same acyl-CoA was used as a cosubstrate. These results suggest that the carbon atom number or charbon chain length of acyl-groups of acyl-CoAs seems to be critical for the 6′-*N*-acylation activity of AAC(6′)-APH(2″). Newly synthesized ISP analogs were isolated from large-scale enzyme reactions and their structures were confirmed by NMR and UPLC-qTOF-HR-MS spectroscopy.

### 3.2. Structural Characterization of New ISP Analogs

To determine the structure of new 6′-*N*-acylated products, A-ISP, P-ISP, and M-ISP, detected in the UPLC-qTOF-HR-MS analysis (Figure 2), each compound was isolated through a cation exchange resin column and a C-18 sample preparation cartridge.

The molecular formula of A-ISP, a pale yellow solid, was determined as C_24_H_45_N_5_O_13_ by the analysis of HR-qTOF-MS data: *m/z* 612.3077 [M + H]^+^ (calculated for C_24_H_46_N_5_O_13_^+^, 612.3087); characteristic fragment ions generated by 4,6-glycosidic link cleavages at [M + H − garosamine (GrsN)]^+^ 453.2208, [1-*N*-AHPA-2-deoxystreptamine (DOS) + H]^+^ 250.1411, [6-*N*-acetyl-aminoglucose (6-*N*-acetyl-aminoGlc) + H − H_2_O]^+^ 204.0866, and [GrsN + H − H_2_O]^+^ 160.0955 (Figure S2A,B). The ^1^H, ^13^C, and HSQC NMR spectra (Figure S2C,D,F, and Table 1) in D_2_O indicated two carbonyl carbons (δ_C_ 173.0; 174.8), two anomer signals (δ_H_ 5.31/δ_C_ 98.2; δ_H_ 5.03/δ_C_ 98.4), two *N*-methylene signals (δ_H_ 3.15 and 3.21/δ_C_ 42.0; δ_H_ 3.36 and 3.43/δ_C_ 39.9), three *N*-methine signals (δ_H_ 4.08/δ_C_ 48.8; δ_H_ 3.42/δ_C_ 48.8; δ_H_ 3.21/δ_C_ 64.6), an *N*-methyl signal (δ_H_ 2.78/δ_C_ 35.3), an oxygenated methylene signal (δ_H_ 4.07 and 3.24/δ_C_ 67.1), nine oxygenated methine signals (δ_H_ 3.68/δ_C_ 80.6; δ_H_ 3.74/δ_C_ 73.1; δ_H_ 3.67/δ_C_ 79.5; δ_H_ 4.35/δ_C_ 67.9; δ_H_ 3.52/δ_C_ 71.3; δ_H_ 3.72/δ_C_ 71.5; δ_H_ 3.61/δ_C_ 72.5; δ_H_ 3.22/δ_C_ 70.6; δ_H_ 3.88/δ_C_ 66.3), an oxygenated quaternary carbon signal (δ_C_ 70.1), a methylene signal (δ_H_ 1.68 and 2.12/δ_C_ 30.5), and two methyl signals (δ_H_ 1.94/δ_C_ 20.6; δ_H_ 1.21/δ_C_ 21.1), displaying a typical feature of ISP with an additional acetyl group. A further interpretation of COSY and HMBC NMR data (Figure S2E,G) revealed that an ISP possesses an AHPA on C1 of DOS, a 6-aminoGlc, a GrsN, and an acetate. The HMBC correlation from a methyl proton (δ_H_ 1.94) of an acetyl group and oxygenated protons (δ_H_ 3.36 and 3.43) to C6′a (δ_C_ 174.8) of A-ISP, respectively, identified the attachment of an acetyl on the amine group at C6′. As expected, the structure of A-ISP was confirmed as a new ISP analog bearing an *N*-acetyl group at C6′.

The second new compound, P-ISP, was isolated as a pale yellow solid with the molecular formula C_25_H_47_N_5_O_13_ given by HR-qTOF-MS: *m/z* 626.3245 [M + H]^+^ (calculated for C_25_H_48_N_5_O_13_^+^, 626.3243); characteristic fragment ions generated by 4,6-glycosidic link cleavages at [M + H − GrsN]^+^ 467.2337, [1-*N*-AHPA-DOS + H]^+^ 250.1379, [6-*N*-propionyl-aminoGlc + H − H_2_O]^+^ 218.1025, and [GrsN + H − H_2_O]^+^ 160.0955 (Figure S3A,B). The ^1^H, ^13^C, and HSQC NMR spectra (Figure S3C,D,F, and Table 1) in D_2_O showed two carbonyl carbons (δ_C_ 173.0; 178.8), two anomer signals (δ_H_ 5.30/δ_C_ 98.0; δ_H_ 5.03/δ_C_ 98.4), two *N*-methylene signals (δ_H_ 3.15 and 3.21/δ_C_ 42.0; δ_H_ 3.36 and 3.43/δ_C_ 39.8), three *N*-methine signals (δ_H_ 4.08/δ_C_ 48.8; δ_H_ 3.42/δ_C_ 48.8; δ_H_ 3.21/δ_C_ 64.6), an *N*-methyl signal (δ_H_ 2.78/δ_C_ 35.3), an oxygenated methylene signal (δ_H_ 4.06 and 3.25/δ_C_ 67.9), nine oxygenated methine signals (δ_H_ 3.68/δ_C_ 80.5; δ_H_ 3.72/δ_C_ 73.1; δ_H_ 3.67/δ_C_ 79.5; δ_H_ 4.34/δ_C_ 67.9; δ_H_ 3.53/δ_C_ 71.3; δ_H_ 3.71/δ_C_ 71.5; δ_H_ 3.61/δ_C_ 72.5; δ_H_ 3.22/δ_C_ 70.6; δ_H_ 3.88/δ_C_ 66.3), an oxygenated quaternary carbon signal (δ_C_ 70.1), two methylene signals (δ_H_ 1.68 and 2.12/δ_C_ 30.5; δ_H_ 2.15/δ_C_ 29.3), and two methyl signals (δ_H_ 0.97/δ_C_ 9.8; δ_H_ 1.21/δ_C_ 21.1). These MS and NMR data revealed that this compound is an ISP analog with an additional propionyl group. A COSY correlation (Figure S3E) between H6′b (δ_H_ 2.15) and H6′c (δ_H_ 0.97) indicated an ethyl residue of additional propionate. In the HMBC spectrum (Figure S3G), a methyl proton (δ_H_ 1.21) and a methylene proton (δ_H_ 2.15) of a propionyl group and oxygenated protons (δ_H_ 3.36 and 3.43) to C6′a (δ_C_ 178.8) of P-ISP, respectively, showed crosspeaks to indicate the attachment of a propionyl on the amine group at C6′. Thus, the structure of P-ISP was defined as a novel ISP analog having an *N*-propionyl group at C6′.

From the analysis of HR-qTOF-MS for M-ISP, which was purified as a pale yellow solid, the molecular formula was given as C_25_H_45_N_5_O_15_: *m/z* 656.2983 [M + H]^+^ (calculated for C_25_H_46_N_5_O_15_^+^, 656.2985); characteristic fragment ions generated by 4,6-glycosidic link cleavages at [M + H − GrsN]^+^ 497.2111, [1-*N*-AHPA-DOS + H]^+^ 250.1400, [6-*N*-malonyl-aminoGlc + H − H_2_O]^+^ 248.0771, and [GrsN + H − H_2_O]^+^ 160.0955 (Figure S4A,B). ^1^H, ^13^C NMR, and HSQC NMR spectra (Figure S4C,D,F, and Table 1) in D_2_O displayed three carbonyl carbons (δ_C_ 172.9; 174.7; 176.7) containing two carbonyl carbons of malonate, two anomer signals (δ_H_ 5.31/δ_C_ 98.4; δ_H_ 5.03/δ_C_ 98.2), two *N*-methylene signals (δ_H_ 3.15 and 3.21/δ_C_ 41.8; δ_H_ 3.36 and 3.43/δ_C_ 39.7), three *N*-methine signals (δ_H_ 4.08/δ_C_ 48.6; δ_H_ 3.42/δ_C_ 48.5; δ_H_ 3.21/δ_C_ 64.4), an *N*-methyl signal (δ_H_ 2.76/δ_C_ 35.1), an *O*-methylene signal (δ_H_ 4.07 and 3.24/δ_C_ 67.7), nine *O*-methine signals (δ_H_ 3.68/δ_C_ 80.4; δ_H_ 3.74/δ_C_ 73.0; δ_H_ 3.67/δ_C_ 79.2; δ_H_ 4.35/δ_C_ 66.8; δ_H_ 3.52/δ_C_ 71.1; δ_H_ 3.72/δ_C_ 71.3; δ_H_ 3.61/δ_C_ 72.3; δ_H_ 3.22/δ_C_ 70.4; δ_H_ 3.88/δ_C_ 66.2), an *O*-quaternary carbon signal (δ_C_ 69.9), two methylene signals (δ_H_ 1.68 and 2.12/δ_C_ 30.3; δ_H_ 1.88/δ_C_ 21.9), and a methyl signal (δ_H_ 1.19/δ_C_ 20.9), revealing a typical feature of ISP with an additional malonyl group. In the HMBC spectrum (Figure S4G), there are crosspeaks from a methylene proton (δ_H_ 1.88) of a malonyl group to C6′a (δ_C_ 174.7) and C6′c (δ_C_ 176.7), and oxygenated protons (δ_H_ 3.36 and 3.43) to C6′a (δ_C_ 174.7), respectively. The correlation of these crosspeaks indicated the attachment of a malonyl group on the amine group at C6′, and the structure of M-ISP was confirmed as a new M-ISP bearing an *N*-malonyl on C6′.

### 3.3. Antibacterial Activity and Cytotoxicity of 6′-N-Acylated ISP Analogs

In general, 1-*N*-acylated AGs including amikacin, arbekacin, ISP, and 1-*N*-6′-*N*-di-acylated AG such as plazomicin were more effective against AG-resistant bacteria compared to their parent drugs [2,4,5]. To test whether the 6′-*N*-acylated ISP analogs have an enhanced antibacterial activity against ISP-resistant pathogens, we conducted drug susceptibility tests with ISP, A-ISP, P-ISP, and M-ISP, following the CLSI guideline. ISP-susceptible and -resistant bacteria (*E. coli* and *P. aeruginosa*) were employed to determine the antibacterial spectra of new analogs (Table 2). The MIC_90_ value of ISP against *E. coli* ATCC 25,922 was determined to be 4 μg/mL, which is slightly higher compared to some of the previously reported MICs. This was probably caused by the larger inoculum size than that used in other reports. It has been reported that some degree of variation in the MIC_90_ values of ISP can be observed depending on the experimental condition [19]. Nevertheless, all newly synthesized ISP analogs displayed significantly decreased MIC values against a total of ten ISP-resistant pathogens compared with ISP in the experiment conducted under the same conditions. Among the compounds tested, M-ISP showed the strongest antibacterial activity against all tested strains. Although A-ISP and P-ISP were less or similarly active against the ISP-susceptible *E. coli* and *P. aeruginosa* compared to ISP, their antibacterial activities against the resistant bacteria were definitely improved. These results imply that the additional *N*-acylation of the C6′-amine position of 1-*N*-acylated AG by AAC(6′)-APH(2″) was effective to circumvent the AG resistance of the tested resistant pathogens, although the detailed resistance mechanism of the test strains is presently unknown.

**Figure 3:**
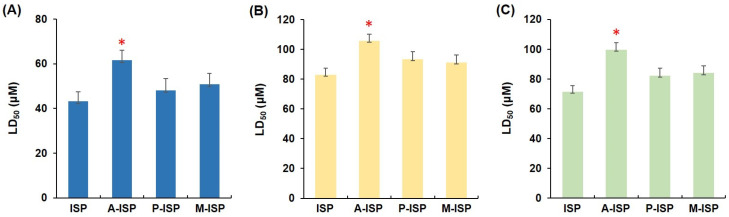
Cytotoxicity of newly synthesized ISP analogs against three different mammalian renal cell lines. LD_50_ (μM) of ISP analogs against (**A**) HEK-293, (**B**) LLC-PK1, and (**C**) A-498 cells. Data are expressed as means (*n* = 3) ± standard deviations and tested for significance using the paired or unpaired two-tailed *t*-test with analysis of variance as appropriate. *n* indicates biologically independent experiments. Results with * *p* < 0.05 were considered significant.

Since the major therapeutic disadvantages of AGs are their high nephrotoxicity and ototoxicity [20], to assess the therapeutic potential of the newly synthesized ISP analogs, their in vitro toxicities were analyzed using three mammalian kidney cell lines HEK-293, LCC-PK1, and A-498. Although the LC_50_ values for three ISP analogs displayed an upward tendency compared to that of ISP in the three cell lines tested (Figure 3), the statistically significant difference in the toxicity profile was only observed with A-ISP: The toxicity of A-ISP was approximately 1.3~1.4-fold lower relative to the ISP control. These results showed that A-ISP which possesses potency against ISP-resistant clinical isolates of *E. coli* and *P. aeruginosa* also has reduced toxicity compared with ISP.

The latest approved semi-synthetic AG plazomicin, which respectively has an AHBA side chain and a hydroxyethyl group at the C1-and C6′-amine positions of the natural AG sisomicin, displays a superior activity against a broad range of Gram-negative pathogens and improves safety in the cytotoxicity profile [5]. It is known that the modified structural motifs of plazomicin block the AG inactivation by clinically relevant AMEs, including AAC(6′), AAC(3), ANT(2″), and APH(2″) [21,22]. Moreover, low levels of nephrotoxicity or ototoxicity of plazomicin were observed in the animal studies [23]. In addition, it has been reported that an installation of the AHBA moiety at the C1-amine position is effective in reducing the toxicity of AGs [24]. Interestingly, the newly synthesized ISP analogs by the simple enzymatic reaction, which were modified in a similar manner to plazomicin, have a potent antibacterial activity against ISP-resistant pathogens with a reduced in vitro nephrotoxicity. Hence, the acylation of the amino groups at C1 and C6′ positions in AG scaffolds appear to be important for the pharmacological properties.

## 4. Conclusions

Natural AGs produced from the soil microorganism actinomycetes are structurally complex molecules, and their regiospecific modifications to overcome the deactivation by AMEs and reduce cytotoxicity remain challenging. Here, we explored the specificity of AAC(6′)-APH(2″) towards ISP and a variety of acyl-CoAs. The substrate promiscuity of AAC(6′)-APH(2″) led to diverse acylations at the C6′-amine position on the ISP scaffold. Newly enzymatically synthesized ISP analogs, A-ISP, P-ISP, and M-ISP, have potent antibacterial activities against the ISP-resistant Gram-negative bacteria tested. Among these analogs, A-ISP exhibited a statistically significant reduction in nephrotoxicity in vitro with an improved antibacterial activity as compared to ISP. These regiospecific structural modifications by the enzymatic synthesis could be used for the generation of novel AG analogs with an improved therapeutic potential. Hence, it is highly likely that these new analogs could be candidates for the future development of AG drugs through further investigation of a resistant mechanism and safety profile.

## 5. Patents

Provisional patent applications covering this work have been filed.

## Figures and Tables

**Table 1:** ^1^H and ^13^C NMR data of novel ISP analogs in D_2_O.

No.	A-ISP		P-ISP		M-ISP	
	δ_C_	δ_H_, (*J* in Hz)	δ_C_	δ_H_, (*J* in Hz)	δ_C_	δ_H_, (*J* in Hz)
1	48.8	4.08 (m, 1H)	48.8	4.08 (m, 1H)	48.6	4.08 (m, 1H)
2	30.5	1.68 (ddd, 15.0, 7.5, 7.5, 1H) 2.12 (brd, 15.0, 1H)	30.5	1.66 (ddd, 15.0, 7.5, 7.5, 1H) 2.12 (brd, 15.0, 1H)	30.3	1.68 (ddd, 15.0, 7.5, 7.5, 1H) 2.12 (brd, 15.0, 1H)
3	48.8	3.42 (m, 1H)	48.8	3.42 (m, 1H)	48.5	3.42 (m, 1H)
4	80.6	3.68 (dd, 7.5, 7.5, 1H)	80.5	3.68 (dd, 7.5, 7.5, 1H)	80.4	3.68 (dd, 7.5, 7.5, 1H)
5	73.1	3.74 (dd, 7.5, 7.5, 1H)	73.1	3.72 (dd, 7.5, 7.5, 1H)	73.0	3.74 (dd, 7.5, 7.5, 1H)
6	79.5	3.67 (dd, 7.5, 7.5, 1H)	79.5	3.67 (dd, 7.5, 7.5, 1H)	79.2	3.67 (dd, 7.5, 7.5, 1H)
1a	173.0		173.0		172.9	
1b	67.9	4.35 (dd, 7.5, 5.0, 1H)	67.9	4.34 (dd, 7.5, 5.0, 1H)	66.8	4.35 (dd, 7.5, 5.0, 1H)
1c	42.0	3.15 (dd, 15.0, 7.5, 1H) 3.21 (dd, 15.0, 5.0, 1H)	42.0	3.15 (dd, 15.0, 7.5, 1H) 3.21 (dd, 15.0, 5.0, 1H)	41.8	3.15 (dd, 15.0, 7.5, 1H) 3.21 (dd, 15.0, 5.0, 1H)
1′	98.2	5.31 (brs, 1H)	98.0	5.30 (brs, 1H)	98.4	5.31 (brs, 1H)
2′	71.3	3.52 (dd, 7.5, 4.0, 1H)	71.3	3.53 (dd, 7.5, 4.0, 1H)	71.1	3.52 (dd, 7.5, 4.0, 1H)
3′	71.5	3.72 (dd, 7.5, 7.5, 1H)	71.5	3.71 (dd, 7.5, 7.5, 1H)	71.3	3.72 (dd, 7.5, 7.5, 1H)
4′	72.5	3.61 (dd, 7.5, 7.5, 1H)	72.5	3.61 (dd, 7.5, 7.5, 1H)	72.3	3.61 (dd, 7.5, 7.5, 1H)
5′	70.6	3.22 (brdd, 7.5, 7.5, 1H)	70.6	3.22 (brdd, 7.5, 7.5, 1H)	70.4	3.22 (brdd, 7.5, 7.5, 1H)
6′	39.9	3.36 (dd, 15.0, 5.0, 1H) 3.43 (brd, 15.0, 1H)	39.8	3.36 (dd, 15.0, 5.0, 1H) 3.43 (brd, 15.0, 1H)	39.7	3.36 (dd, 15.0, 5.0, 1H) 3.43 (brd, 15.0, 1H)
6′a	174.8		178.8		174.7	
6′b	20.6	1.94 (s, 3H)	29.3	2.15 (q, 8.0, 2H)	21.9	1.88 (s, 2H)
6′c			9.8	0.97 (t, 8.0, 3H)	176.7	
1″	98.4	5.03 (brs, 1H)	98.4	5.03 (brs, 1H)	98.2	5.03 (brs, 1H)
2″	66.3	3.88 (dd, 7.5, 4.0, 1H)	66.3	3.88 (dd, 7.5, 4.0, 1H)	66.2	3.88 (dd, 7.5, 4.0, 1H)
3″	64.6	3.21 (d, 7.5, 1H)	64.6	3.21 (d, 7.5, 1H)	64.4	3.21 (d, 7.5, 1H)
4″	70.1		70.1		69.9	
5″	67.1	4.07 (brd, 15.0, 1H) 3.24 (brd, 15.0, 1H)	67.9	4.06 (brd, 15.0, 1H) 3.25 (brd, 15.0, 1H)	67.7	4.07 (brd, 15.0, 1H) 3.24 (brd, 15.0, 1H)
6″	35.3	2.78 (s, 3H)	35.3	2.78 (s, 3H)	35.1	2.76 (s, 3H)
7″	21.1	1.21 (s, 3H)	21.1	1.21 (s, 3H)	20.9	1.19 (s, 3H)

**Table 2:** Antibacterial activity of ISP and its analogs.

Bacterial Strains	MIC_90_ (µg/mL)			
	ISP	A-ISP	P-ISP	M-ISP
*E. coli* ATCC 25922	4	8	4	2
AREC ^a^ P00538	32	16	16	4
AREC ^a^ P00579	>64	16	16	8
AREC ^a^ P00650	>64	8	16	4
AREC ^a^ P00651	>64	8	8	4
AREC ^a^ P00661	32	16	8	4
*P. aeruginosa* ATCC 27853	8	16	8	4
MDRPA ^b^ 1–21	>64	8	16	4
MDRPA ^b^ 1–23	>64	16	16	4
MDRPA ^b^ 1–67	>64	16	8	2–4
MDRPA ^b^ 2–22	>64	16	16	2
MDRPA ^b^ 2–35	>64	32	16	4

^a^ Antibiotic-resistant *E. coli*; ^b^ multi-drug resistant *P. aeruginosa*.

## Supplementary Materials

The following are available online at https://www.mdpi.com/2218-273X/10/6/893/s1, Figure S1: Coomassie blue-stained SDS-PAGE gel of purified C-terminal hexahistidine tagged AAC(6′)-APH(2″); Figure S2: The structural determination of 6′-*N*-acetyl isepamicin; Figure S3: The structural determination of 6′-*N*-propionyl isepamicin; Figure S4: The structural determination of 6′-*N*-malonyl isepamicin.
