# Supplementary material for: Development of 6′-N-Acylated Isepamicin Analogs with Improved Antibacterial Activity against Isepamicin-Resistant Pathogens
Source: Biomolecules. 2020 Jun 11;10(6):893. doi: 10.3390/biom10060893 (PMC7356214; doi:10.3390/biom10060893)
Supplement: Supplementary file 1 [file biomolecules-10-00893-s001.pdf]

Supplementary Materials

Development of 6′-*N*-acylated isepamicin analogs with improved antibacterial activity against isepamicin-resistant pathogens

Yeon Hee Ban [1], Myoung Chong Song [1], Hee Jin Kim [2], Heejeong Lee [3], Jae Bok Wi [4], Je Won Park [4], Dong Gun Lee [3], and Yeo Joon Yoon [1,*]

[1] Natural Products Research Institute, College of Pharmacy, Seoul National University, Gwanak-gu, Seoul 08826, Republic of Korea; yhban@snu.ac.kr (Y.H.B.); smch517@snu.ac.kr (M.C.S.)

[2] Department of Chemistry and Nanoscience, Ewha Womans University, Seoul 03760, Republic of Korea; kimijini93@gmail.com (H.J.K.)

[3] School of Life Sciences, BK21 Plus KNU Creative BioResearch Group, College of Natural Sciences, Kyungpook National University, Daehakro 80, Bukgu, Daegu 41566, Republic of Korea; gml09wjd@naver.com (H.L.); dglee222@knu.ac.kr (D.G.L.)

[4] Department of Integrated Biomedical and Life Sciences, Korea University, Seoul 02841, Republic of Korea; slwlwhs@korea.ac.kr (J.B.W.); jewonpark@korea.ac.kr (J.W.P.)

* Correspondence: yeojoonyoon@snu.ac.kr (Y.J.Y.); Tel.: +82-2-880-2379 (Y.J.Y.)

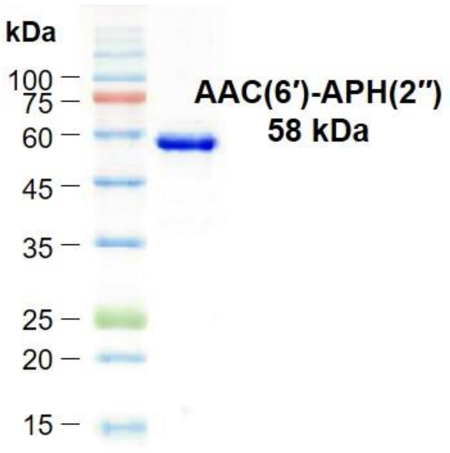

Figure S1. Coomassie blue-stained SDS-PAGE gel of purified C-terminal hexahistidine tagged AAC(6′)-APH(2″).

(A)

(B)

(C)

(D)

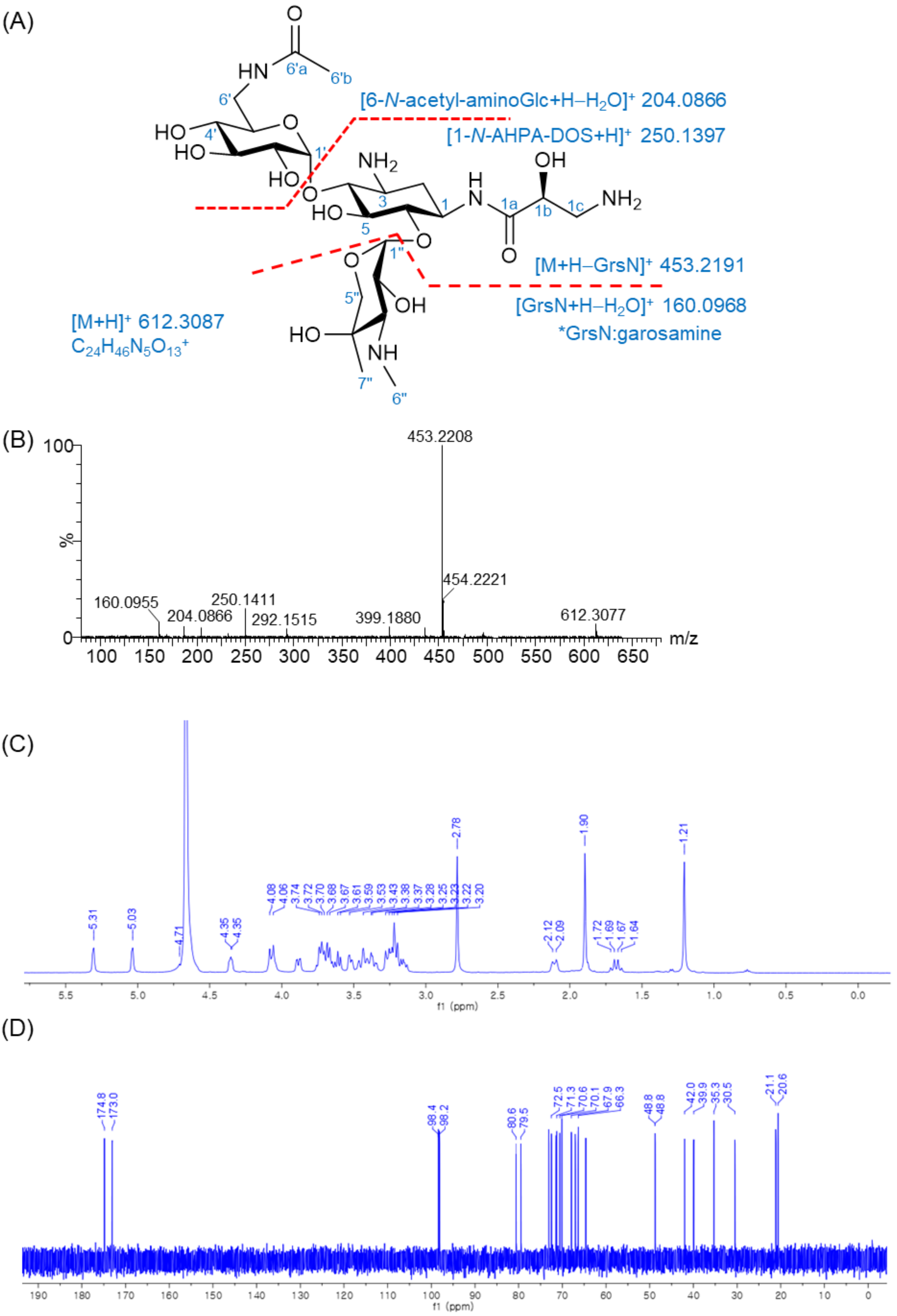
[6-N-acetyl-aminoGlc+H–H2O]+ 204.0866
[1-N-AHPA-DOS+H]+ 250.1397
[M+H–GrsN]+ 453.2191
[GrsN+H–H2O]+ 160.0968
*GrsN:garosamine
[M+H]+ 612.3087
C24H46N5O13+
453.2208
454.2221
160.0955
204.0866
250.1411
292.1515
399.1880
612.3077
m/z
%
f1 (ppm)
5.31
5.03
4.71
4.35
2.78
1.90
1.21
174.8
173.0
98.4
98.2
80.6
79.5
48.8
42.0
39.9
35.3
30.5
21.1
20.6

(E)

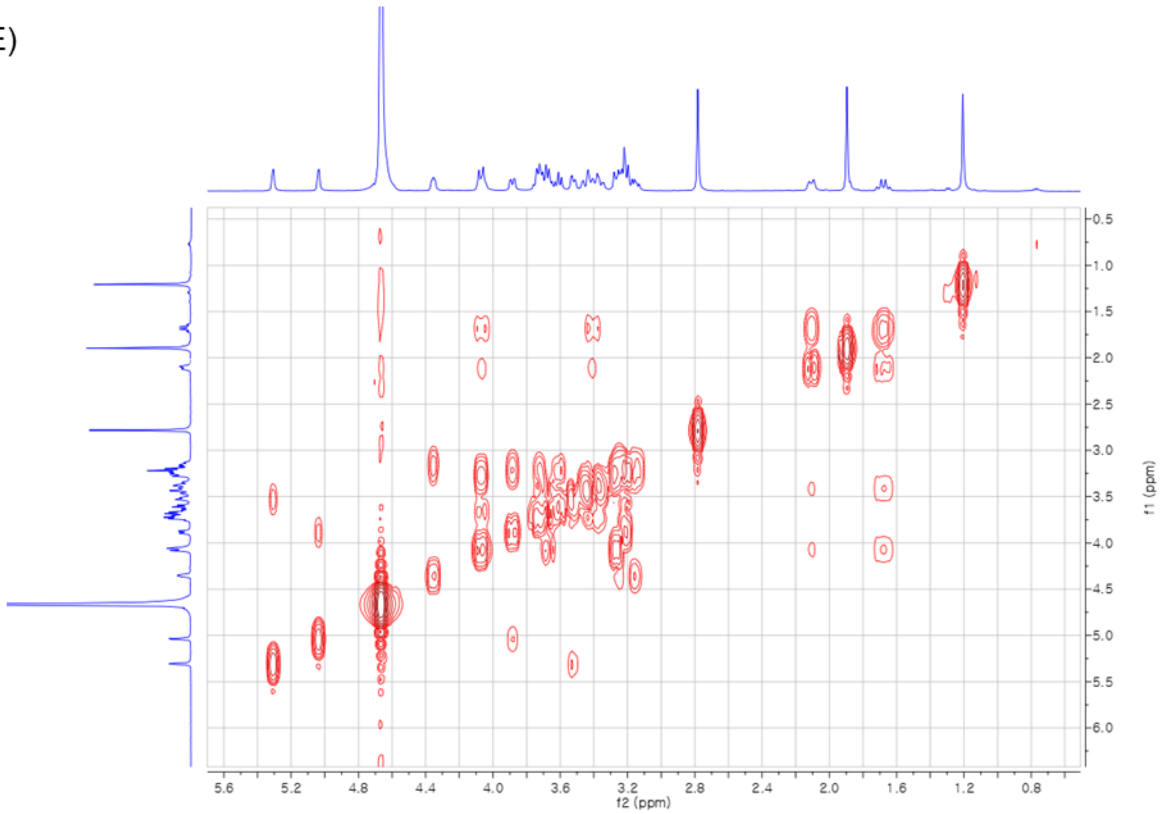
5.6
5.2
4.8
4.4
4.0
3.6
3.2
2.8
2.4
2.0
1.6
1.2
0.8
f2 (ppm)
0.5
1.0
1.5
2.0
2.5
3.0
3.5
4.0
4.5
5.0
5.5
6.0
f1 (ppm)

(F)

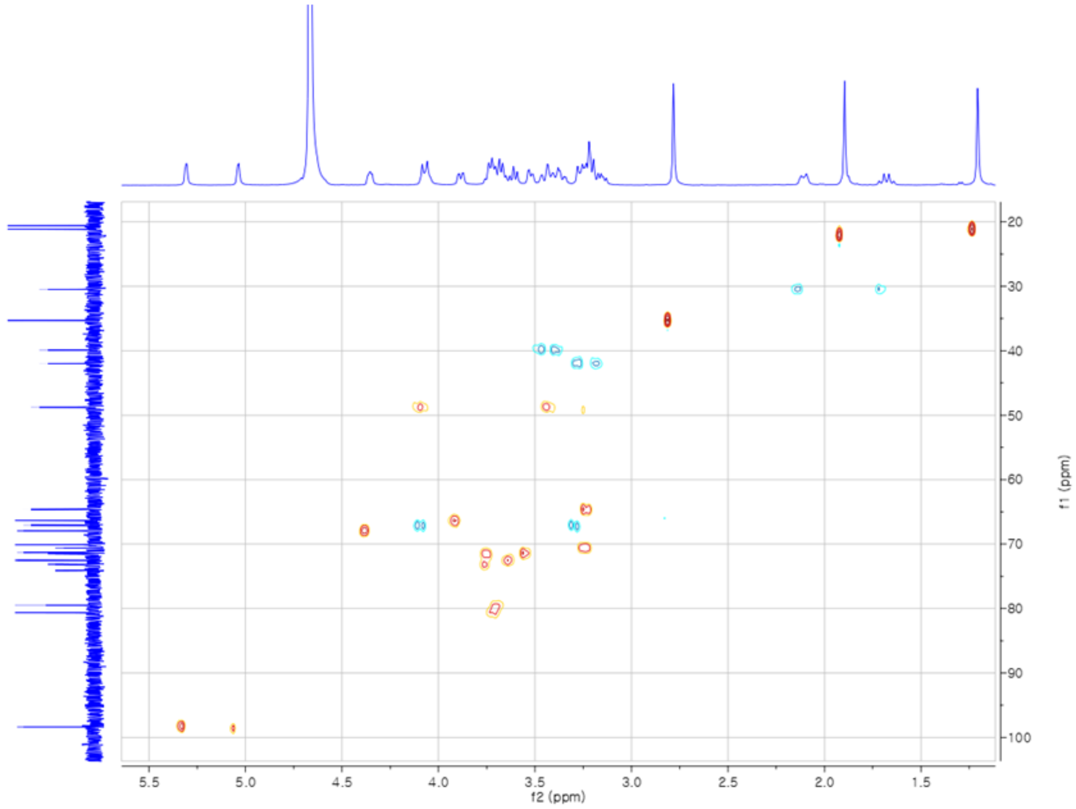
5.5
5.0
4.5
4.0
3.5
3.0
2.5
2.0
1.5
f2 (ppm)
20
30
40
50
60
70
80
90
100
f1 (ppm)

(G)

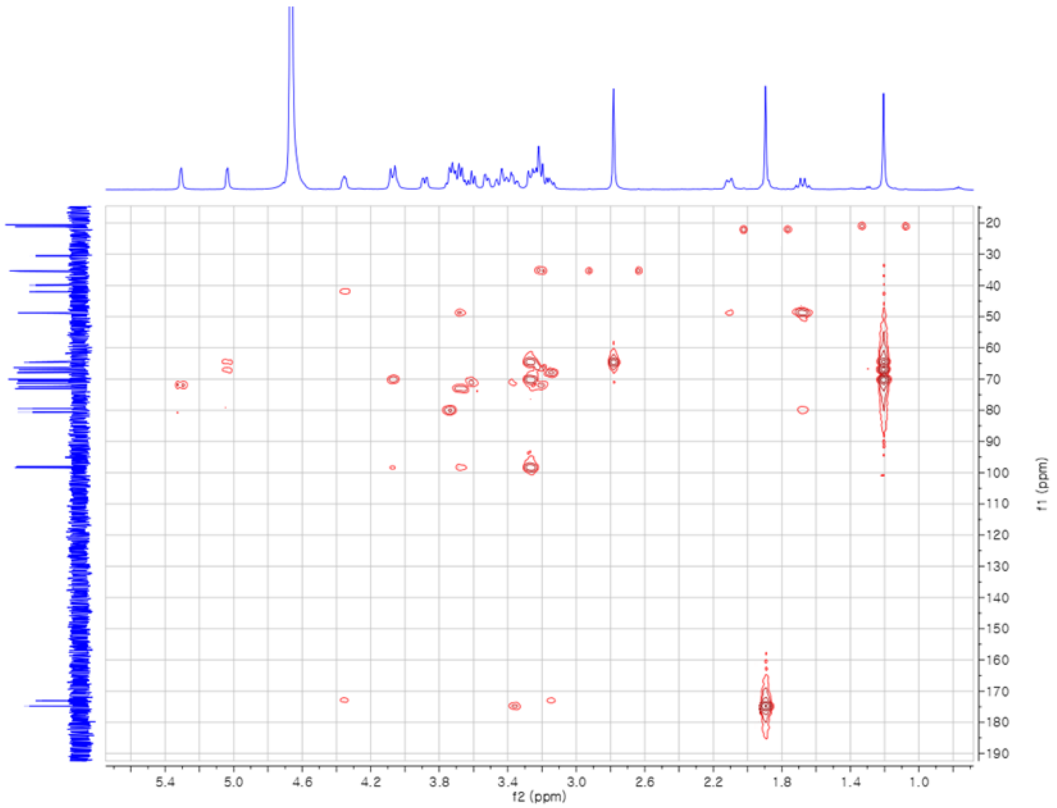

Figure S2. The structural determination of 6′-*N*-acetyl isepamicin. (A) MS/MS fragmentation pattern (B) MS/MS spectra (C) ^{1}H NMR data (D) ^{13}C NMR data (E) ^{1}H-^{1}H gCOSY NMR data (F) ^{1}H-^{13}C gHSQC NMR data (G) ^{1}H-^{13}C gHMBC NMR data

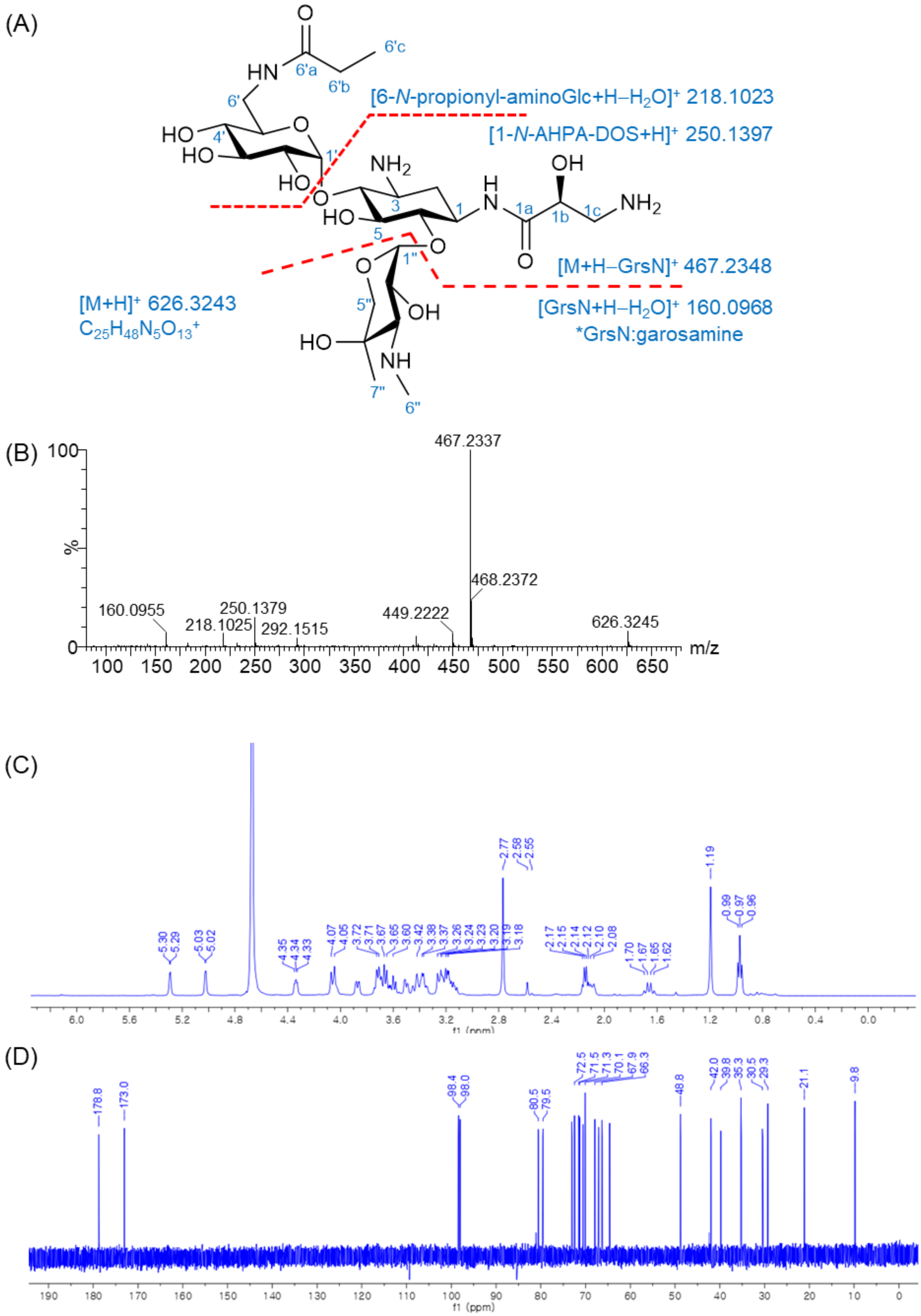
(A)
[6-N-propionyl-aminoGlc+H–H2O]+ 218.1023
[1-N-AHPA-DOS+H]+ 250.1397
[M+H–GrsN]+ 467.2348
[GrsN+H–H2O]+ 160.0968
*GrsN:garosamine
[M+H]+ 626.3243
C25H48N5O13+
(B)
467.2337
468.2372
160.0955
250.1379
218.1025
292.1515
449.2222
626.3245
%
m/z
(C)
f1 (ppm)
(D)
f1 (ppm)

(E)

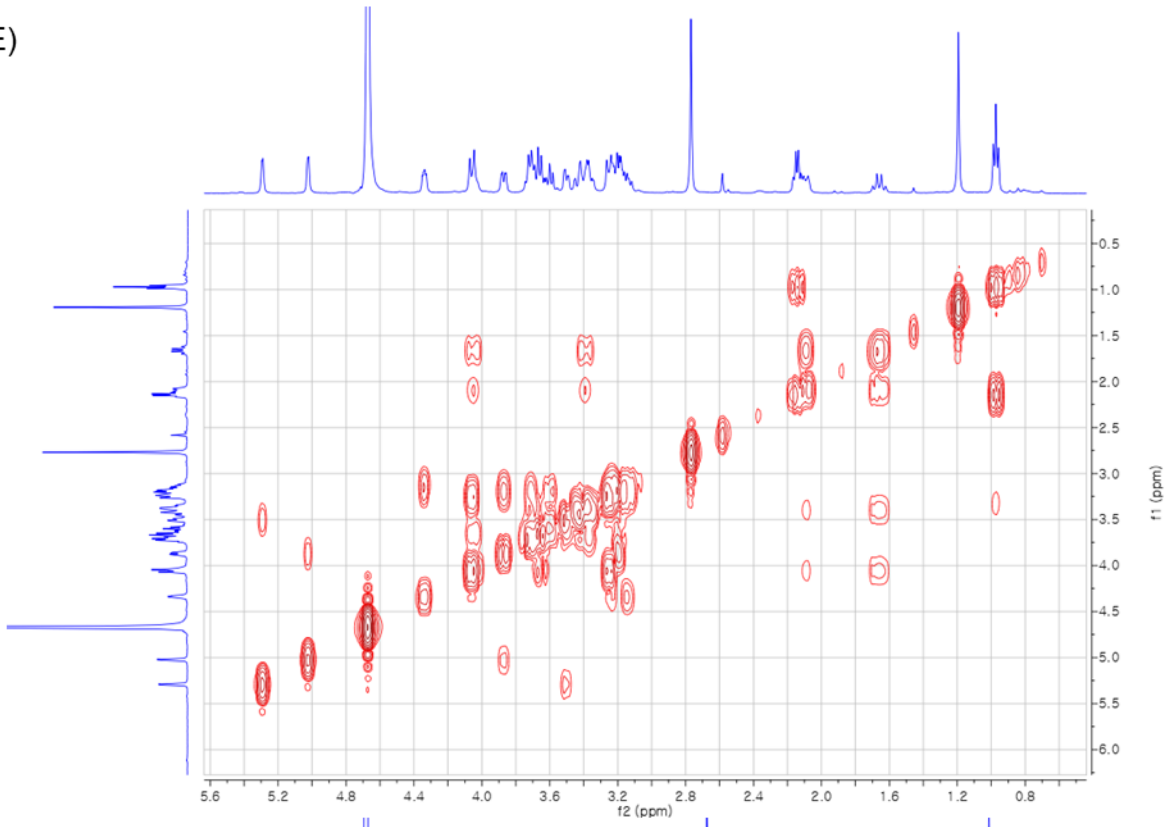

(F)

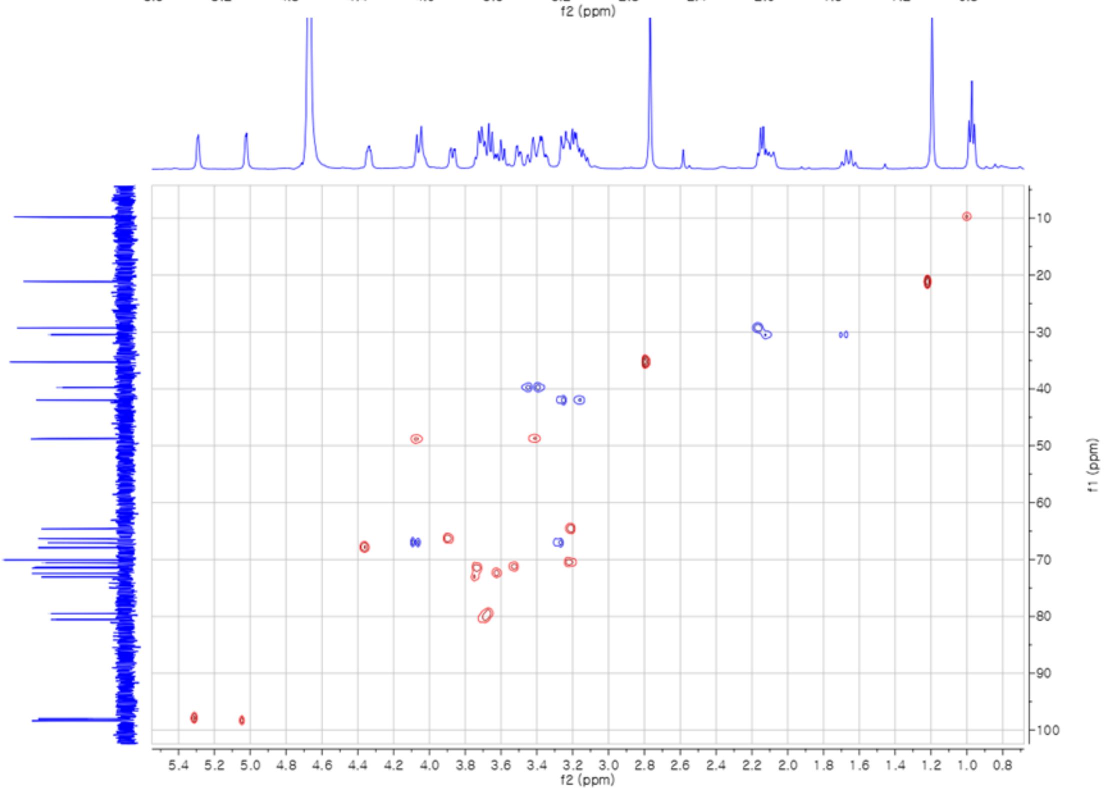

(G)

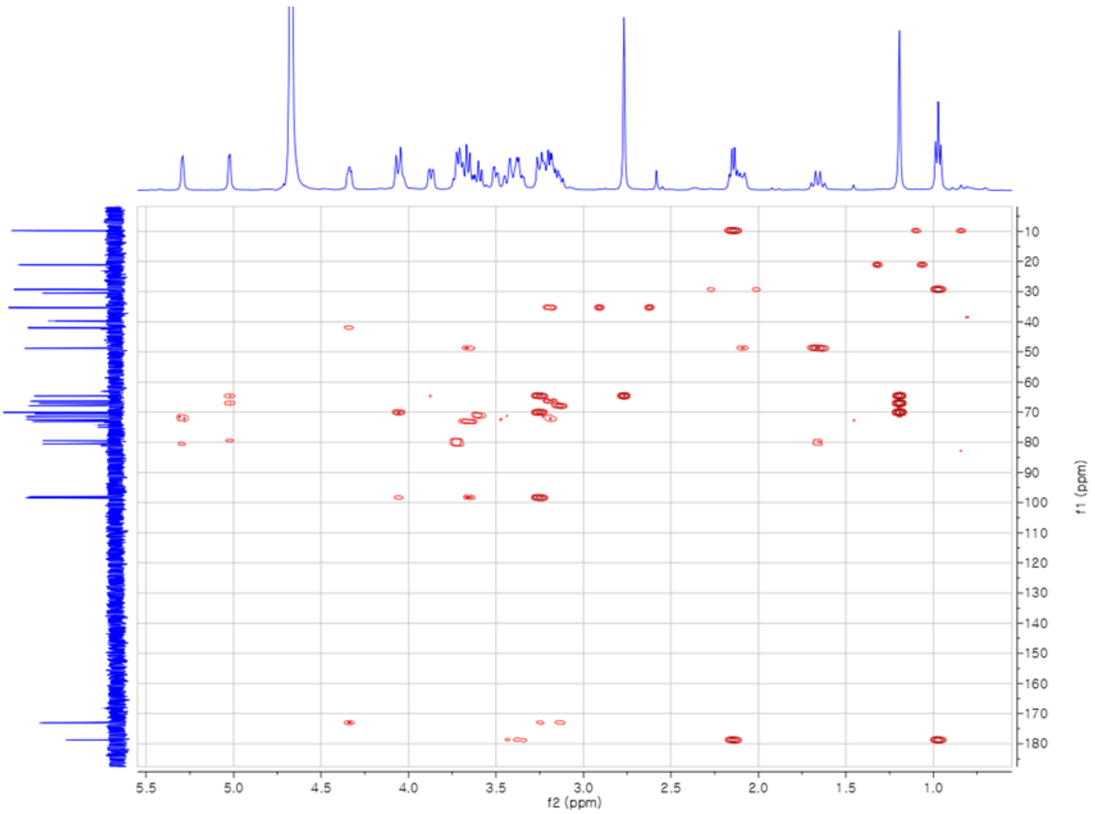

Figure S3. The structural determination of 6′-*N*-propionyl isepamicin. (A) MS/MS fragmentation pattern (B) MS/MS spectra (C) ^{1}H NMR data (D) ^{13}C NMR data (E) ^{1}H-^{1}H gCOSY NMR data (F) ^{1}H-^{13}C gHSQC NMR data (G) ^{1}H-^{13}C gHMBC NMR data

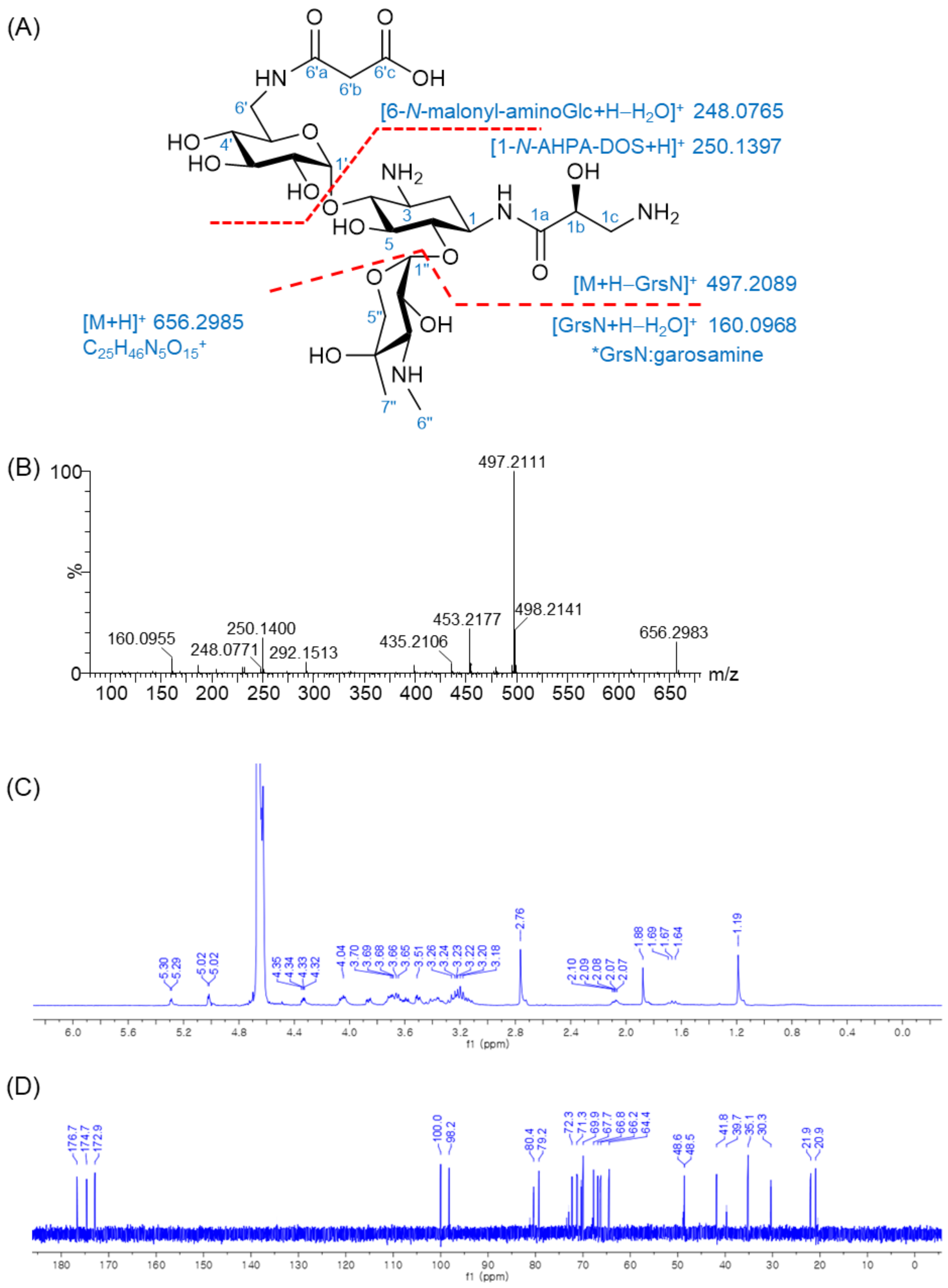
(A)
HN
6'a
6'b
6'c
OH
6'
HO
4'
HO
HO
1'
NH2
OH
H
N
1a
1b
1c
NH2
3
1
HO
5
1"
5"
OH
HO
NH
7"
6"
[6-N-malonyl-aminoGlc+H–H2O]+ 248.0765
[1-N-AHPA-DOS+H]+ 250.1397
[M+H–GrsN]+ 497.2089
[GrsN+H–H2O]+ 160.0968
*GrsN:garosamine
[M+H]+ 656.2985
C25H46N5O15+
(B)
100
%
0
497.2111
498.2141
453.2177
656.2983
435.2106
250.1400
160.0955
248.0771
292.1513
m/z
(C)
5.30
5.29
5.02
5.02
4.35
4.34
4.33
4.32
4.04
3.70
3.69
3.68
3.66
3.65
3.51
3.26
3.24
3.23
3.22
3.20
3.18
2.76
2.10
2.09
2.08
2.07
2.07
1.88
1.69
1.67
1.64
1.19
f1 (ppm)
(D)
176.7
174.7
172.9
100.0
98.2
80.4
79.2
72.3
71.3
69.9
67.7
66.8
66.2
64.4
48.6
48.5
41.8
39.7
35.1
30.3
21.9
20.9
f1 (ppm)

(E)

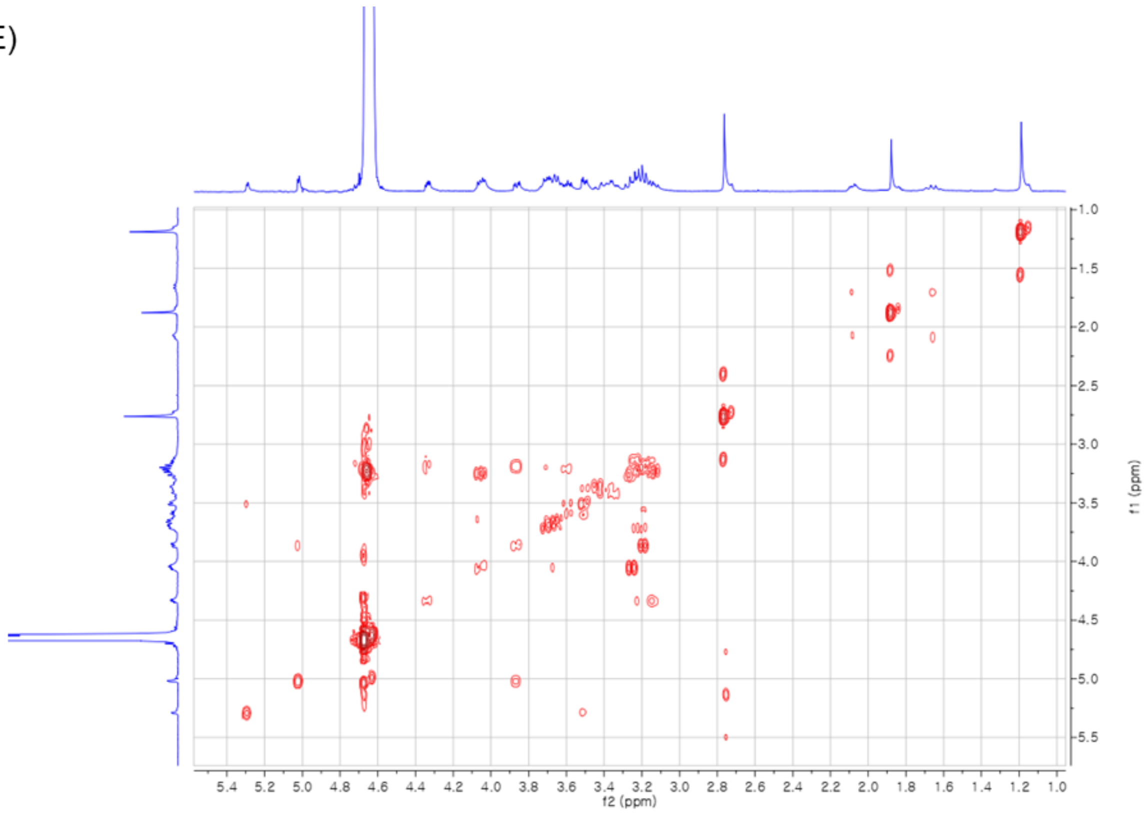

(F)

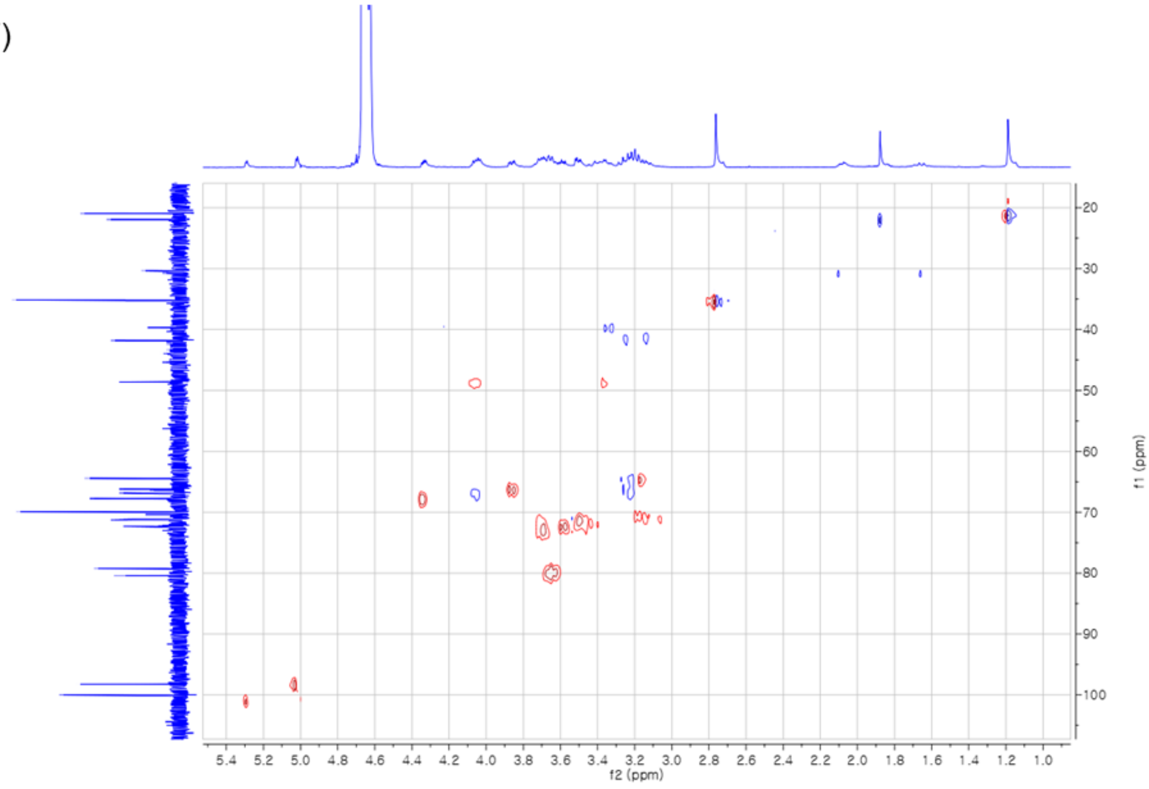

(G)

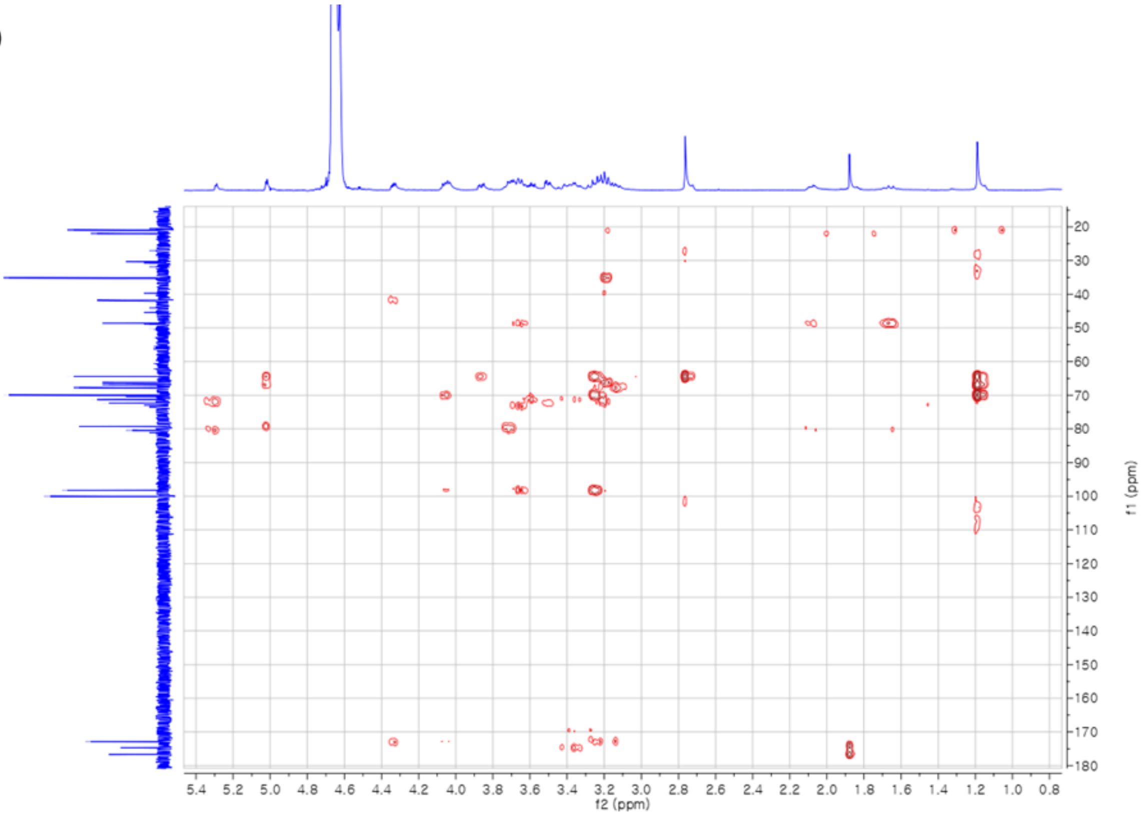

Figure S4. The structural determination of 6′-*N*-malonyl isepamicin. (A) MS/MS fragmentation pattern (B) MS/MS spectra (C) ^{1}H NMR data (D) ^{13}C NMR data (E) ^{1}H-^{1}H gCOSY NMR data (F) ^{1}H-^{13}C gHSQC NMR data (G) ^{1}H-^{13}C gHMBC NMR data
